# Contour Fillings.—Has Experience Demonstrated Their Practical Value

**Published:** 1874-06

**Authors:** H. L. Sage

**Affiliations:** Bridgeport, Conn.


### SELECTED ARTICLES.

---

### ARTICLE IV.

### *Contour Fillings.—Has Experience Demonstrated their Practical Value.*

BY H. L. SAGE, D. D. S., BRIDGEPORT, CONN.

* * * * The word *contour*, as applied in operative dentistry, may possess different shades of meaning, and should be strictly defined. It is a French Word, and signifies in its original sense, "the outline; the line that bounds, defines or terminates a figure."

Therefore, as relating to a tooth, it would designate the line that defines the shape or bounds the peripheral portion in any individual case—and contour filling, the restoration, by any material in use for the purpose of such, when lost by disease, abrasion or accident; which restoration also includes the bulk or body of the lost portion, or rather the substitution *for* such, of any material which will best answer the purpose.

The sense is restricted, first: to a tooth which, having lost its entire crown, has it restored (in the sense in which the substitution of one material for another constitutes a restoration) by a substance, which, for most practical purposes, will take the place of the original tooth structure; always supposing that substance to be gold, as being the best material yet discovered or employed for the purpose.

Secondly: to a filling in or restoration of contour of a tooth which, having partially lost its crown, a whole or a portion of the walls of the cavity has been broken down, or rendered so thin and friable as to necessitate the trimming of the borders. This may include a sixteenth, (or less,) an eighth, a quarter, a third, a half, three-quarters, or even more of the crown—and may usually include either of the proximal surfaces, partially the grinding or the cutting edge.

Thirdly: to the filling and restoration of contour of a tooth, bereft of either of its palatine (or lingual) or buccal surface, as the case may be, including also one or both of the proximal—partially or wholly—reference being made more particularly to the bicuspids and molars.

Fourthly: to the substitution of gold for the lost portions of incisor and cuspid teeth, when the same are worn away by occlusion on the palatine surface—a substitution not only to prevent further wear, but maintain perfect antagonism or occlusion.

Fifthly: to the restoration (not to be accurate as to terms) of a class of teeth, as the incisors and canines—perhaps the bicuspids to some extent, though rarely—in which the caries has progressed so far as to involve a considerable amount of the labial surface as well as the proximal and a portion of the palatine—leaving, for instance, only a cylinder of dentine enclosing the pulp, or outwardly only the cutting edge, and a narrow portion of the palatine surface intact.

The latter class is, I imagine, the one most frequently, though erroneously considered as unworthy of an attempt to remedy by filling.

The first designated class—an entire crown of gold built upon a root—will usually include the filling of the latter, though not always, for cases are presented in which the vitality of the pulp may be maintained and yet sufficient anchorage secured to make the operation feasible and permanent, without necessitating the destruction of the pulp. For example, a gold crown was inserted upon a left superior lateral incisor root for a lady patient, Feb. 21st, 1871. This is in good condition and serviceable, though stability was insured without the aid of screws. Furthermore, the pulp is alive and apparently healthy.

Even this class of work will stand the test of time and wear of mastication, if it is a perfect operation of its kind; and many cases in practice will show that a gold crown, even if built upon a lateral incisor root, inferior or superior, will do good service for years, though secure anchorage upon one of these is more difficult to obtain than is the case with larger teeth.

For instance, the roots of an inferior sixth year molar, left side, with the caries extending to the alveolar process on the posterior proximal portion, were prepared for the reception of a gold crown. After properly adjusting the rubber dam, four and one-third dwts. of gold, (light and heavy) six hours of malleting, and five and one-half of preliminary preparation and finishing, sufficed to furnish a substitute for the only absent crown on this side of the mouth, which substitute has been a source of comfort to the individual from that time (three years ago) to this, and is likely to remain so many years longer—at the same time stimulating the antagonizing tooth by keeping it in healthful activity.

A projecting central incisor (superior) which had been broken off ten years, was subjected to treatment and placed in a condition to tolerate a restoration of crown.

A root filling of gold seven lines in length was inserted. An entire gold crown was also built upon the root continuously with the filling in the latter, and the natural projection entirely overcome, thus placing the gold crown regularly in the arch. Artistically, it presented a fine appearance, with a compact, mirror-like surface and perfect restoration of contour, by dint of mallet force and proper shaping and finishing. Three dwts. of gold and nine hours' time, aside from treatment, sufficed to render the tooth efficient. A gold crown anchored in a root canal seven lines in length ought to insure permanence, and doubtless will. Perfect adaptation at the base, firm borders, secure anchorage, a densely impacted filling, etc., will not disappoint such an expectation. Many cases might be cited, but this is unnecessary.

It is not always well to put the highest polish on a highly finished crown, though a very smooth surface is essential. A dead finish may be better, but if the young man requests a high polish, it is well to produce it, and if his companions give him the sobriquet of the "little light house," as was done in the above case, the gold can be rendered less conspicuous by a little labor, *i. e.*, by reducing the luster. Much depends upon the conspicuousness which one naturally gives to the teeth in speaking or laughing.

Where a number of gold crowns are required in the same mouth it is good practice to insert them, as my experience has demonstrated by numerous cases in practice.

The principal objection to a gold crown in the anterior part of the mouth is its conspicuousness or unnatural appearance. But this one disadvantage cannot outweigh the more positive advantages of comfort, cleanliness, efficiency in mastication, ease in articulation, the preservation of the root and its maintenance in a wholesome condition, prevention of absorption of alveolus, and the support which an unimpaired alveolar arch and the retention of the roots therein implanted gives to the adjoining teeth, especially when the fangs are in health, so far as, when dead, they may be, and mounted with serviceable gold crowns—that they may also receive the stimulus which an organ in a state of activity receives from the performance of its functions—more especially when some degree of vitality is maintained—aside from the fact that the natural contour of the features is preserved, which, when lost, artificial substitutes can never fully restore.

Another important consideration is the absence of that annoyance which the wearing of a plate often produces, to say nothing of the positive injury, more or less, to the gums and remaining teeth which it (a partial artificial denture) causes, be it ever so nicely adapted to the mouth, or composed of innocuous materials—producing, as it does, a greater or less degree of irritation or inflammation of the mucous membrane, absorption of the soft and hard tissues, and consequent loosening of the natural organs, and also vitiation of the fluids of the mouth, which, acting upon the teeth, become productive of caries.

These consequences are sometimes dependent upon the wearing of artificial teeth, though in a modified degree in many cases—but when rubber is the material employed, how great is its influence for evil. Rubber has its place, and is useful in many directions in the arts, but as a base for artificial teeth it is more injurious than useful.

When gold is substituted for the lost natural tooth structure, so as to constitute an entire restoration of contour, and the root maintains its vitality, or if dead, is placed in a wholesome condition before proceeding with the operation—such a substitute cannot, so far as all the before mentioned advantages are concerned, be distinguished by the happy possessor from the adjoining teeth.

Did any one ever hear of a patient wishing to be rid of such a tooth, if all the necessary preliminary steps were taken and the operation made as perfect as it is the duty and privilege of an honest and skillful operator to make it? Rarely, if ever.

Pivot teeth, though *looking* natural, are, as *usually* inserted, not durable, but very objectionable and unsatisfactory—far more so than a well-finished gold crown ever is or can be—while the former cannot be adapted to the roots of the large grinding teeth or the two-fanged bicuspids; but with gold restoration can be had, and be made permanent and very useful in mastication.

A pivot tooth does not prevent a root from decaying or becoming abscessed. It is true that the roots of incisors and canines can be preserved by the insertion of certain expensive kinds of pivot teeth, but somehow patients will rarely bear the expense of procuring them, but will usually be better satisfied to pay a reasonable price for the gold crown, with its one objection—not very conspicuous after all—unnatural appearance. But why sacrifice the advantages pertaining to this class of work to natural appearance?

The appearance is but slightly unnatural, and the gold is usually mostly hidden from view. Teeth discolored by caries present a *natural* appearance, for naturally they decay and if let alone a natural death is the consequence. Such decaying and blackened teeth are annoying, injurious, filthy and offensive both to the eye and to good taste, and many are so far destroyed as to necessitate the gold showing, if they are filled at all properly, it being nearly as conspicuous in many cases as if the entire crown were of this material, even if but one-quarter be restored, or the borders merely protected by overlapping.

Yet who would sacrifice a tooth on this account, or forego the benefit of entire restoration of contour?

Which are preferable, finely finished contour fillings, or cavities with friable, jagged borders, dark-looking, semicircular spots in the crowns of teeth, revealing with every smile the consequences of neglect?

The expression is certainly uninjured by the former, and the natural contour of the lip preserved, while if one or more teeth are lost, a plate with teeth attached will not as fully restore it, but will render the speech less natural, modifying the tone of the voice and preventing that full and free circulation which a full natural dentine insures.

As to certain modifications of the gold crown—a gold crown with a porcelain face for instance—my experience will not warrant an opinion, nor does it enter into the subject under consideration.

A few words on the second class of contour fillings—fillings constituting a partial restoration of contour, in which is involved one and sometimes both proximal surfaces, in which the walls of the cavity are so broken down, or thin and fragile, as to necessitate trimming them with file or chisel until firm and square borders are secured.

This in the incisors and canines may also involve the cutting edge its entire width, or a fraction of it, or in a bicuspid or molar a whole or a part of the grinding surface. In an incisor, the portion lost by decay and filing and shaping the borders, may represent labio-proximally and palato-proximally a crescent shaped cut, leaving the corner of the cutting-edge intact, or more frequently, it will be necessary to sacrifice this portion and restore with gold. In any case the outline of the cut at the cervical portion will present a curve.

Teeth frequently come to our notice where the trimming cannot be avoided without sacrificing the beauty and permanence of both tooth and contour filling.

When the loss of any portion of the labio-proximal or palato-proximal surface of an incisor or cuspidatus becomes necessary, the border should be at least protected by overlapping with gold, and the same may be said of the bicuspids or molars when the corresponding surfaces of these are involved. But it is better that the restoration should be entire than partial, particularly with the front teeth, for a space between these of any considerable extent not only renders pronunciation more difficult, but mars the appearance; and as to conspicuousness, a partial restoration in these cases is, as before stated, more objectionable than full, showing the gold quite as plainly in the one case as in the other, with loss of contour in the former to mar the symmetry and offend the eye by an indefiniteness of form or shape. A V-shaped space is entirely unnecessary with these teeth, for decay here is not likely to recur if the operation is perfect and the patient pays any attention to cleanliness.

But that some discrimination should be exercised in regard to cases in which contour fillings, so-called, are or are not applicable, is apparent, considered with regard to full and partial restoration. For instance, in the case of very much crowded bicuspids of a peculiar shape—straight-sided teeth perhaps they may be appropriately designated—teeth as wide, or nearly, across the buccal or palatine (or lingual) surfaces, *i. e.*, from surface to surface proximally, at the cervical portion, as they are at, near or toward the grinding surface, in contradistinction from a tooth with the crown diameter proximally much the shortest at the neck.

One objector says contour fillings afford pockets at the necks of the teeth, and thus from the difficulty of keeping them clean, or because they are not self-cleaning, caries is produced at the base of filling, a result of the lodgment and retention of food and secretions.

But the trouble with these straight or parallel-sided teeth is, that if, when they are much crowded or the jaw is full, they are restored to their original shape, they *do not* afford pockets, and therefore are with difficulty cleansed, and hence are more likely to decay at the base of the filling than teeth with narrow necks.

Again, when a tooth is isolated, or standing a little apart from its neighbor there should be a full restoration of contour when lost, whatever may be the conformation. In teeth with the mesial and distal surfaces in contact the entire length of their crowns, or nearly, entire restoration of contour when lost is not usually advisable, as they are then cleansed with difficulty, if at all, and caries is more or less liable to recur. In such a case, a V-shaped space should be left, only protecting the filed borders by neatly over-lapping with as much gold as will be sufficient for the purpose; and where the insertion of ordinary fillings, or fillings including the proximal and grinding surface becomes necessary in this class of straight-sided and crowded teeth, it is often more conducive to their preservation, especially if they are of poor quality, to cut away enough of the tooth to render it self-cleansing; otherwise the most thorough and expert operator even, will be pained to find, in some cases, and after a few years, a recurrence of caries at the base of a well inserted filling.

In the other class, (long and slim bicuspids with narrow necks) the loss should be made good by full restoration—the sides only touching at a point near the grinding surface as in nature, so that all foreign matter may be easily removed with pick and brush from about the filling at the base, and from between the teeth at the necks. These pockets then are more advantageous than otherwise, for they render the teeth capable of being more easily cleansed; but we all know how difficult this becomes when there is no space between them, and then, as before stated, decay is very likely, sooner or later, to return, especially with the bicuspid teeth, with which more failures occur than with the incisors or canines, by far, or the molars, if we except poorly formed sixth-year.

Aside from these considerations, full restoration of the grinding surface should be made, when the V-shaped space is not imperatively demanded, for the efficiency of a tooth is greatly lessened, and mastication of the food rendered an uncomfortable operation when any considerable portion of the grinding surface of one or more teeth is absent, to say nothing of the irritation of the gums which is often caused by the wedging of food in the space made vacant by the loss of tooth substance by caries or by having been cut away by the advocates of the Arthur system of treatment. But the filling should be cut down and shaped so as to maintain perfect antagonism and not prevent the adjoining teeth from shutting together properly, as well as not to hazard the permanence of the filling and render the tooth in which it is inserted sore from irritation produced by contact of the antagonizing tooth with an unnatural fullness of the restored grinding surface.

But it is not my purpose to enter into details with regard to methods of manipulating.

A well-inserted contour filling can, in many instances perhaps, be subjected to considerable rough usage without injury.

A clergyman received by accident a severe blow on the lip, with such force as to produce a slight indentation in the face of a well impacted contour filling in the right superior cuspid. This tooth has been dead seventeen years, having, as long ago, received a root-filling but having only a quarter of the crown remaining at the upper portion, full restoration of contour was made four years ago.

But two dwts. of gold and five hours' time sufficed to make good what would be to a clergyman, especially, a serious loss and inconvenience. Neither the filling or the tooth received any injury, nor was the adaptation of the gold to the latter disturbed in the least. The teeth in this mouth are of the finest quality, very hard and dense.

In another case the results were more serious. A lady had a central incisor with a loss by caries of two-thirds of the crown, involving the labio and palato-proximal surface and two-thirds of the width of the cutting edge. Full restoration of contour was made six years ago, including a root filling. The crown received a severe blow from the elbow of a companion, filling the natural portion with numerous fractures, radiating like the spokes of a wheel, and breaking off the golden corner of the cutting edge. Notwithstanding, the mechanical union of the filling with the tooth remained perfect. The tooth was naturally soft and fragile, but the loss was repaired by building on the broken portion, since which time ( March 9, 1870 ). it has remained as before.

Now, by the existence of the labor-saving burring engine, the time in preparing cavities and cutting down or shaping and finishing the palatine and grinding surfaces of fillings, is materially shortened; while by the use of the mallet, handled by an assistant, the gold may be so thoroughly welded and condensed as to render the thin cutting edges of incisors when restored secure against anything but violent blows, as in the cases instanced above.

The first filling of this kind ( a partially restored incisor crown ) with which any personal experience was had, soon broke down from a want of practice with this specific kind of operative dentistry. But success crowned subsequent efforts, the mallet being used in all cases without exception.

Hard rubber, lead or similar metals, and steel were in turn employed—the latter being finally adopted as being the best and most effectual for the purpose, and the least unpleasant to patients.

But restoration of contour is applicable, not only to the front teeth, but to inferior and superior bicuspids and molars, not excepting in favorable cases, the *dentes sapientæ*.

In regard to the third class—bicuspids requiring a restoration of contour from having lost a palatine ( or lingual ) or buccal surface, including of necessity some of the proximal—doubts have been entertained and expressed as to the durability of contour fillings in teeth with cavities of this specific class. But here there is as much danger of the remaining buccal or palatine wall of the tooth breaking down in mastication as that the filling will give way—though if the masticating surface is properly shaped and not too full, so that the antagonizing tooth will not act as a wedge, thus forcing and spreading the gold against the remaining natural wall or cusp of the tooth until it is fractured, there is little to apprehend from this source, and the time spent in restoring such teeth to their original shape is well occupied; for the success may be as complete as with any other class of contour fillings, with the chances for permanence also, nearly as great, provided all the essential details which belong to thorough and skillful operating are complied with.

With the molars a large surface and a sufficiency of room for secure anchorage is especially favorable to success.

In the fourth class of contour fillings the incisors and cuspidati are restored when worn away by occlusion or abrasion, as it is called. This may be spontaneous or mechanical, but here reference is made to the latter, ( though the remedy is the same in either case ) usually caused by the loss of the back teeth, when all the force of occlusion is distributed to the anterior part of the mouth, thus making the palatine a grinding surface and necessitating the substitution of gold for the lost tooth structure, in order to prevent further abrasion, preserve vitality, if not lost, and render the teeth more efficient in mastication.

The palatine surface may not only be included in the loss, but where the teeth strike perpendicularly together—the cutting edges meeting—the crowns may be worn away the entire diameter of their axes. Usually, however, the labial wall will be standing—wholly or partially intact. That is, the anterior and posterior palato-proximal and the palatine surfaces will always be involved, and sometimes, when the case is complicated with caries, the anterior and posterior labio-proximal surfaces.

All will admit the practical value of restoration in cases as described, if perfect antagonism is maintained, and firmly anchored fillings of cohesive foil are solidly impacted with mallet force.

In cases like the foregoing the extensive loss of dentine is usually followed by absorption and recession of pulp tissue, produced by the aggression of the antagonizing tooth upon the territory of the latter; but the diminished pulp often retains its life by throwing out for its covering and protection a deposit of secondary dentine—the abrasion producing just enough healthy inflammatory action for the purpose.

The fifth and last class of cavities requiring a restoration of contour, and a substitute for the portion lost by decay, merit more than a passing notice, because such, especially when any considerable number are involved, would, by the majority, probably be counted as worthless and past remedy. But such teeth, and the necessary operations to be performed, can be best described and illustrated by citing a case in practice, to which reference has before been made, as an example of the ravages of caries in a mouth in which the secretions were excessively alkaline, and also of the effect of these upon Guillois' Cement with which some of them were at first filled. (See *Dental Register*, July, 1872, Vol. XXVI, page 59.)

In these teeth cavities were present in almost every conceivable location, not excepting the lingual surfaces of the inferior right and left, first and second molars—the cavities running transversely and groove-like, just about the margins of the gums. But the six superior front teeth had deep and nearly crescent-shaped cavities extending on the labial surfaces, to and above the margins of the gums, and downward to such an extent as to give the appearance of being, when filled—gold crowns with enameled cutting edges.

The anterior and posterior labio and palato-proximal angles were also involved, leaving in some cases, a narrow neck of enamel on the palatine surface at the narrowest portion separating the two ends of the two-third or three-quarter circles of gold, with which the teeth were encompassed. Thus each pulp was protected by a mere thin covering of dentine, for none were exposed though the cavities were deep; and the young man might with propriety have exclaimed in the words of Job, "I am escaped with the skin of my teeth."

During the operation of filling these teeth the rubber dam was employed, as in the majority of cases it should be, and though it seemed impossible to adjust it perfectly and satisfactorily to the labial surface of a root, above a cavity, where the same extended much above the margin of the gum, even in this particular location it afforded material aid, as by keeping it on a stretch at this point, it acted as a compress and kept the gums, which were then unhealthy, from exuding those peculiar, viscid, and almost imperceptible secretions, which often prove so annoying without the dam is employed. Aside from this little difficulty sometimes met with, its perfect adaptation to the tooth otherwise, is attended with great benefit during operations on other parts of a cavity of this description.

Wedges were also introduced between the teeth, and assisted greatly in keeping the dam in place. Here they are indispensable.

Thus the fillings were kept dry during every operation, perfect adaptation of the gold to the borders of the cavities was secured, which rendered them air and moisture-tight—so that every filling is as it should be—faultless; and presents a compact, flush, smooth and mirror-like surface—in fact a restoration of contour. The time and material employed, as to amount in each case, ranged as follows:

Right central incisor—time, $5\frac{1}{4}$ hours—gold 1 dwt. 8 grs.

Left central incisor—time, $6\frac{3}{4}$ hours—gold, 1 dwt. 12 grs.

Right lateral incisor—time, $7\frac{3}{4}$ hours—gold, 1 dwt.

Right cuspidatus—time, $7\frac{3}{4}$ hours—gold, 1 dwt. 6 grs.

Left lateral incisor—time, $5\frac{1}{2}$ hours—gold, 22 grs.

Left cuspidatus—time, $5\frac{1}{2}$ hours—gold 2 dwts.

The left inferior canine is also filled in the same manner.

Subsequently the gums received proper treatment, and not being longer subjected to the irritation which they once received from overlapping the jagged edges of the cavities, are now hard and healthy.

These teeth were pronounced past remedy five years ago, and the question would be asked by many, will this class of operations pay, so far as durability is concerned—bounded as the fillings are on the cervical borders by cementum? perhaps the reply would be in the negative, even though strict regard was had to cleanliness by the patient.

Will not caries again recur at the line of junction of the cementum and the filling?

The fillings in the above case were inserted during the past year, and therefore sufficient time has not elapsed to determine with any certainty as to the recurrence of caries at the margins of the gums, but the probability is that cleanliness will prevent this.

This may be considered an exceptional case in practice, considering the destructive type of the disease, or the magnitude of the loss and restoration, and the time, labor and skill necessary to be employed, and the difficulties to be overcome in the introduction of the gold.

If perfect operations will insure success the preservation of these teeth is certain. The life of the pulp was in every case maintained.

These fillings might not be strictly defined as contour as the term is usually applied, but as the restoration of the greater part of every surface was included in most of them, the term would seem appropriate.

It would seem that caries would be likely to attack the healthy cementum borders of cavities bounded by the latter, even if the fillings were perfect, and cleanliness were maintained; but as the cementum has more vitality than the enamel, and more direct sources of nourishment, receiving it directly from both pulp and periosteum, this might serve to overbalance the danger which would ensue when exposed, from its having a greater amount of organic constituents than other parts of the tooth.

But when cementum becomes exposed it is not in a normal condition, and is not therefore in a state to resist disease, as it would be if protected by its natural covering. Neither is the dentine, when unprotected.

But the latter is sometimes enabled to check the further progress of caries, by a deposition of ossific matter in the tubuli, thus rendering it harder and denser, and less capable of being influenced by the action of chemical agents—and perhaps the cementum is subject to the same law of defensive warfare.

But this is a question to be determined by those best capable of judging.

If the fluids of the mouth were excessively alkaline the cementum would be more likely to suffer than if the reverse were true, containing as it does more organic matter than the enamel and a little more than the dentine, or according to an analysis by Lassaigne, nearly one-half, as will be seen by the following table:

| | | |
|---|---|---|
| Organic matter . . . . . | | 42.18 |
| Phosphate of lime . . . | 53.84 | |
| Carbonate of lime . . . | 3.98 | 57.82 |
| | | 100.00 |

Von Bibra, however, makes the cementum to contain:

| | |
|---|---|
| Organic matter . . . . . | 29.27 |
| Earthy matter . . . . . . | 70.73 |
| | 100.00 |

Making the chemical composition of the dentine and cementum of nearly the same proportion.

Alkalies seize upon the animal portions of the teeth and remove them, and thus produce caries, as seems to have been the effect in this case. This class of cavities, and what was done in this instance has been partially explained and described, and yet more fully than any other, in order to direct attention to the question: What are the chances in favor of the permanent effects of such treatment in caries of the teeth, of which the last described has been presented as a specific type, form or example?

And here I would rest the matter by asking your indulgence for the unintentional length of this paper, which it has seemed quite difficult to confine to narrow limits, without taking more time than could be consistently devoted thereto, and which is requisite in order to canvass a subject in all its bearings, and yet retain only the pith or essence or the full meaning by a condensation of matter.—*Missouri Dental Journal.*

